# Biogas production using the microbial community present in the soil from Deception Island, maritime Antarctica

**DOI:** 10.1007/s11356-026-37814-9

**Published:** 2026-05-20

**Authors:** Patricia Yolanda Alderete Ortigoza, Franciele Natividade Luiz, Giovanna Juliana Ghellere, Rafaela Faust Meyer, Luiz Henrique Rosa, Michel Rodrigo Zambrano Passarini

**Affiliations:** 1 Laboratório de Biotecnologia Ambiental, Universidade Federal da Integração Latino-Americana (UNILA), Av. Tarquínio Joslin dos Santos, 1000 – Jd Universitário, Foz do Iguaçu, PR 85870-650 Brazil; 2 Centro Internacional de Energia Renovável (CIBIOGAS-ER), Itaipu, Foz do Iguaçu, PR Brasil; 3 Departamento de Microbiologia, Universidade Federal de Minas Gerais, Belo Horizonte, MG Brasil

**Keywords:** Cold microbial communities, Anaerobic digestion, Metagenomic, Methane, Oat, Thawing soil

## Abstract

The current energy crisis is increasing the production of sustainable energy, such as biogas, a fuel generated by the anaerobic digestion of organic waste. The use of oat, an agricultural waste, makes the anaerobic digestion more sustainable. Antarctic microbial communities can utilize a wide range of substrates and adapt to different temperatures. Thus, this study evaluated methane production through an innovative approach, using microbial enrichment, and assessed archaeal diversity through metagenomic techniques in Antarctic soils, Deception Island, Maritime Antarctica. Metagenomic analyses showed low archaeal diversity and abundance. The Euryarchaeota (95.2%) and *Methanobrevibacter* were the most abundant and frequent phylum and genus, respectively. The average biogas production values ​​were 595 LN kg VS⁻^1^ and 561 LN kg VS⁻^1^ in tests with individual oat (IO) and oat with enriched mixed culture (O + MC), respectively. However, O + MC showed a higher methane production, 4% (319 LN kg VS⁻^1^) more than the results from the IO test with inoculum. Soils from Deception Island may represent a promising source of methanogenic communities capable of producing methane using agricultural waste as an alternative for energy production. Future studies are needed to understand the methane production using soil samples from cold environments.

## Introduction

The world has been facing an environmental problem due to the increasing greenhouse gas (GHG) emissions. Carbon dioxide (CO_2_) and methane (CH_4_) are the main components of these gases (Vishnivetskaya et al. 2018). In this way, there is a great need to develop sustainable practices to reduce emissions of these gases in all sectors of the economy.

Anaerobic digestion (AD) is a process used to treat organic waste and produce renewable energy, mainly methane (CH_4_) (Aguilar-Muñoz et al. 2022)_._ This involves four distinct stages, including hydrolysis, acidogenesis, acetogenesis, and methanogenesis (Bonugli-Santos et al. 2022; Mlaik et al. 2022; Jameel et al. 2024). Methane is produced in the methanogenic phase through the transformation of byproducts from previous steps, such as acetic acid, CO_2,_ and H_2_, by methanogenic archaea (Anwar et al. 2021; Luiz et al. 2023). The use of agricultural waste to make AD more sustainable and reduce its impact is a current strategy.

Oat is a lignocellulosic substrate, rich in cellulose, hemicellulose, and lignin. It has a complex structure and is difficult to degrade (Kusch et al. 2011). Brazil is the fourth largest producer of oats, with an output of 1.17 million tons between 2023 and 2024. This large amount of waste generated in the country can be used for biogas production (USDA 2024). These characteristics make the residues of this plant material an important substrate for use as a more friendly and sustainable energy source.

During the AD, when temperatures are close to 37 °C, biogas production can be affected. AD processes are conducted within a mesophilic range (Aguilar-Muñoz et al. 2022; Van et al. 2020). The energy required to operate anaerobic digesters in regions with temperate to cold climates at mesophilic temperatures represents around 30% of the biogas produced (McKeown et al. 2009a). The use of a psychrophilic or low-temperature-adapted microbial consortium can ensure stable biogas production; therefore, the choice of inoculum is a key factor for better viability of anaerobic digestion at low temperatures (McKeown et al. 2009a, b).

In cold regions of the planet, a significant amount of organic carbon can be stored in frozen soils. Almost all frozen organic matter is composed of plant remains, with half of this material found in the first 3 m of permafrost soil. With the increase in greenhouse gases and consequent rise in global warming, these frozen soils begin to thaw. Thus, more organic matter is made available for soil microbial communities to decompose this carbon into more GHGs, including CO_2_ and CH_4_ (Ottoni et al. 2022). Permafrost soils are characterized by microbial communities comprising bacteria, archaea, yeasts, and cyanobacteria that can survive and thrive for thousands of years under extreme conditions of low temperatures and oligotrophic soil (Burkert et al. 2019; Collins and Margesin 2019).

Bacterial communities are more abundant in these environments; however, the phyla Euryarchaeota and Crenarchaeota are the most predominant from the domain Archaea (Collins and Margesin 2019; Steven et al. 2009). The methanogenic potential and the use of Antarctic soils for CH_4_ production have been little studied (Aguilar-Muñoz et al. 2022). In this way, the use of microbial communities found in cold environments can be a sustainable strategy for improving biogas production in AD processes. Thus, the present study evaluated methane production using an innovative approach, employing microbial enrichment from soil collected on Deception Island, Antarctica, and oat as an agricultural residue. A methanogenic approach was used to characterize the archaea community associated with the Antarctic soil.

## Materials and methods

### Soil sampling

Soil sample in the process of thawing was collected during the Austral summer of 2022 on 12/12/2022 at Whalers Bay, Deception Island, Antarctica (−62° 58′ 44.0″ S, 60° 33′ 20.8″ W). Fifty grams of thawed soil were sampled in triplicate (150 g) from a hill, at a depth of approximately 5 cm, using a sterilized spatula, and placed in triplicate in sterilized Whirl–Pak plastic bags (Nasco, USA). The samples were stored at −20 °C in sterile containers until processing, according to Passarini et al. (2024) Fig. 1.

**Fig. 1:**
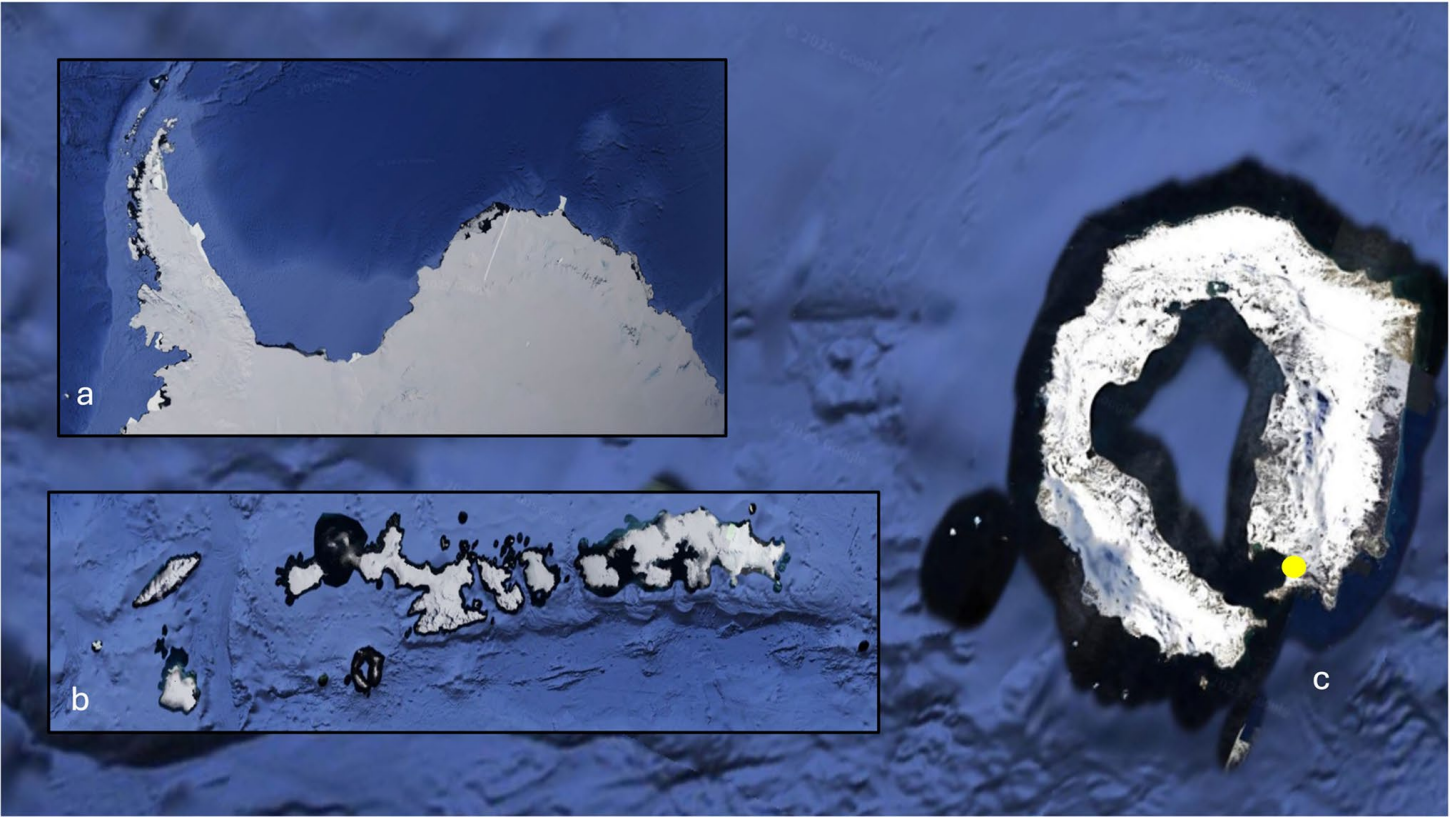
Google maps image (2025) of the soil sample collection site. **a**) Antarctic Peninsula; **b**) South Shetland Islands; **c**) Whalers Bay, Deception Island (yellow dot indicates collection site)

### Oat processing and physical chemical assay

The oat used was obtained from customs inspection and sent to the Biogas Laboratory of the International Center for Renewable Energy—Biogas (CIBiogás), accredited by ABNT NBR ISO/IEC 17.025. Oats were crushed using a Britania Diamante 800 blender. The characterization of Individual Oat (IO) was performed by Total Solids (TS), Fixed Solids (FS), and Volatile Solids (VS) according to the Standard Methods for the Examination of Water and Wastewater (Rice et al. 2017), using the gravimetric method number 2540-B.

The oat samples were processed in porcelain crucibles calcined in a muffle at 550 °C. The samples were dried in an oven at a constant temperature of 104 °C for 12 h. Then, samples were weighed on an analytical balance. Assays were performed in duplicate. The calculated values were following Eq. (1):

1 $$\mathrm{TS}\%=\frac{\mathrm P2-\mathrm P1}{\mathrm{PA}-\mathrm P1}\times100$$

The FS determination was performed using the same crucibles containing the sample after the TS determination. The crucibles were calcined again in a muffle at 550 °C for 4 h. The crucibles were weighed again until a constant value was reached. The FS and VS values were obtained following Eqs. (2) and (3) (Rice et al. 2017):

2 $$\mathrm{FS}\%=\frac{\mathrm P3-\mathrm P1}{\mathrm P2-\mathrm P2}\times100$$

3 $$\mathrm{VS}\%=\frac{\mathrm P2-\mathrm P3}{\mathrm P2-\mathrm P1}\times100$$

where:

P1 = weight of the empty crucible (g).

PA = initial weight of the oat sample (g).

P2 = weight of the crucible after drying the samples (g).

P3 = weight of the crucible after calcining the samples (g).

### Enrichment preparation (mixed culture—MC) using soil from Deception Island

The enrichments were prepared using two liquid culture media (40 mL) with the addition of 5 g of soil: *a)* simple medium, for oligotrophic organisms (M1) containing: peptone 0.25 g L^−1^ and glucose 0.1 g L^−1^; and *b)* nutrient-rich medium (M2) containing: meat extract 1.0 g L^−1^; yeast extract 2.0 g L^−1^; peptone 5.0 g L^−1^; sodium chloride 5.0 g L^−1^. Controls of the same culture medium were used, without the addition of soil. The flasks were sealed with cotton plugs and aluminum foil. The flasks were incubated at 28 °C (due to the climatic conditions of the volcanic Deception Island) for 15 days. The assay was performed with 5 replicates of each medium. After 15 days, the liquid culture media M1 and M2 were mixed to form MC. Denser solid particles present in the MC, originating from the soil sample, were separated by decantation. The liquid phase with microbial growth was used in the BMP assay. MC samples were used with an inoculum (comprising methanogenic microorganisms) and were subjected to the BMP assay (VDI 4630 2016).

### Inoculum

The inoculum from the company CiBiogás was a sludge composed mostly of swine waste and cattle manure, cultivated in an anaerobic reactor with a system operated discontinuously and controlled temperature (37.5 ± 2 °C), pH (7.5), and constant stirring. Daily, the inoculum received a diet consisting of carbohydrates, proteins, and lipids. To maintain inoculum productivity, it periodically receives an organic load made up of a mixture of 70% swine effluent and 30% cattle manure.

### Biochemical methanogenic potential (BMP) assay

#### Anaerobic digestion equipment

The anaerobic digestion equipment consisted of a 250 mL glass digester, where the inoculum and substrate were added. A glass tube graduated from 0 to 500 mL, known as an eudiometer, was attached to this digester, which had a barrier liquid inside that served as an indicator (VDI 4630 2016). The barrier liquid was a solution composed of Na_2_SO_4_.10H_2_O and H_2_SO_4_ 99%, in a 7:1 ratio. Drops of methyl orange were added to the barrier liquid, which, in contact with acid, changed color to pink (Faulverhaltens 1985).

The eudiometer was connected to a compensating bottle, which served as a storage of the barrier liquid when it was displaced by the gas produced in the system, through a system of communicating vessels. The assay was performed in a system operated discontinuously, with a temperature of 37.5 ± 2 °C controlled by a water bath system. The assays were performed in triplicate. Substrate and inoculum masses to be used in the BMP assay were calculated, before incubation, according to VDI 4630 standard (VDI 4630 2016). For this, Eq. (4):

4 $$\frac{\mathrm{V}\mathrm{S} \,\mathrm{o}\mathrm{f}\,\mathrm{s}\mathrm{u}\mathrm{b}\mathrm{s}\mathrm{t}\mathrm{r}\mathrm{a}\mathrm{t}\mathrm{e}}{\mathrm{V}\mathrm{S}\, \mathrm{o}\mathrm{f} \,\mathrm{i}\mathrm{n}\mathrm{o}\mathrm{c}\mathrm{u}\mathrm{l}\mathrm{u}\mathrm{m}}\le 0.5$$

The assay was performed in 6 flasks (2 triplicates) as follows: *i)* triplicate 1 containing: individual oat and inoculum (IO); *ii)* triplicate 2 containing: IO, 30 mL of MC, and inoculum (O + MC). As a negative control, the inoculum was incubated individually. The efficiency of the inoculum's biological activity was evaluated through the incubation of a sample of microcrystalline cellulose, which is recognized as a reference substrate with an estimated potential range for biogas production between 740 and 750 LN kg VS⁻^1^, considered a positive control (VDI 4630 2016). The masses of oat and microcrystalline cellulose were calculated in relation to the amount of inoculum used (200 g), in each proportion of 1:3 (S/I), using Eq. (5) (VDI 4630 2016):

5 $$\mathrm{Sample}\;\mathrm{mass}\;\left(\mathrm g\right)=\frac{\mathrm m}{\%\mathrm{VS}}\times100$$

where:

m = mass (g) of VS on a wet basis of the inoculum (200 g) divided by the determined proportion (1:3).

% VS = percentage of VS on wet sample basis.

The values of the masses and the real volume (mL) of the MC are shown in Table 1. The data were presented as the average of the set of values; therefore, was accompanied by their standard deviation values. To ensure the anaerobic condition, nitrogen gas was added to the eudiometers. The volume of gas generated was recorded daily by observing the eudiometer graduation. The gas composition was determined daily, using a Landtec/Geotech model GA 5000 Multigas analyzer.

**Table 1:** Mass values used in the Biochemical Methanogenic Potential (BMP) assay

Sample	Predicted mass of substrate (g)	Real mass of substrate (g)/SD	Mass of inoculum (g)/SD	Mixed culture (MC) (mL)/SD
Negative control (inoculum)	-	-	203.3 ± 4.25	-
Positive control (Microcrystalline cellulose)	1.40	1.38 ± 0.005	205.9 ± 0.80	-
Individual oat	1.53	1.53 ± 0.005	212.2 ± 14.8	-
O + MC	1.53	1.59 ± 0.08	202.5 ± 1.50	30

O + MC: oat + mixed culture

-: not used

SD: standard deviation

The specific methane production was calculated using the values of VS present in the samples (VDI 4630 2016). The sample methane yield (IO), the methane production value of the negative control (negative), was calculated by subtracting the average methane production of the studied triplicates. The pH conditions, VFA concentration, and alkalinity of the inoculum were monitored through the analysis of the FOS/TAC (free organic acids and total inorganic carbon) coefficient, an indicator for maintaining the stability of the process, as it helps in determining the concentration of VFA and total inorganic carbon of the inoculum (VDI 4630 2016). Samples were kept in batches for 31 days.

### Statistical analysis

Standard deviation was calculated using Excel. Analysis of variance was performed using PAST software version 4.03 (Rani et al. 2022), considering p-values and F-values, with a significance level of p < 0.05 and approximately 95% confidence, using One-way ANOVA, Kruskal–Wallis, and Dunn's post hoc test.

### Metagenomic DNA sequencing

Metagenomic analyses were performed by Passarini et al (2024). About 0.25 g of soil was extracted using the DNeasy Powersoil Pro (Qiagen) according to the manufacturer's recommendation. Metagenomic analysis was performed by a specialized company located in Toledo, Paraná State, Brazil. The metagenomic libraries were prepared using the Nextera XT kit V2. Amplicon sequencing was performed on the Illumina MiSeq platform with a paired-end run. was used to assess the quality of the raw sequencing data.

The sequencing data were analyzed using FastQC software (Wingett and Andrews 2018) to assess the quality of the raw sequences. Subsequently, the sequences were processed using Trimmomatic software (Bolger et al. 2014) to remove low-quality. The data were submitted to the MG-RAST (MetaGenome Rapid Annotation using Subsystem Technology, v3.1) server for annotation (http://metagenomics.nmpdr.org). Annotation of the assembled contigs was performed using BLAST against the Silva database.

## Results and discussion

### Physicochemical analysis

About 88.78% of total solids (TS) was detected in the oat sample, in accordance with Dubrovskis and Plume (2017), who detected 90.17% TS in the oat sample. This difference may vary due to the variations in substrate composition or the presence of other plant parts. Approximately 4.94% TS were observed in the O + MC sample, representing an additional 1.53 g of oat. In the MC sample, TS content was 0.65%, less than 1%, and did not interfere with the amount of biomass used (Table 2).

**Table 2:** Characterization of total, volatile, and fixed solids, and biogas and methane production

Sample	Mass (g)	Volume (mL)	TS (%)	VS (%)	FS(%)	Biogas (LN/Kg VS⁻^1^)/SD	Methane (LN/Kg VS⁻^1^)/SD	Methane (%)
IO	1.53	-	88.78	96.58	3.42	595 ± 17.9	315 ± 7.5	53
MC	-	30	0.65	13.2	86.8	-	-	-
O + MC	1.53	30	4.94	86.11	13.89	561 ± 26.5	319 ± 0.0	57

IO: individual oat

MC: mixed culture

O + MC: oat + mixed culture

TS: total solid

VS: volatile solid

FS: fixed solid

- not determined

SD: standard deviation

Approximately 3.42% of FS was detected in the oat sample. Dubrovskis and Plume (2017) detected 5.84% FS in their study, a value higher than that of non-biodegradable solids, suggesting a difference in sample composition. In O + MC, an increase in the FS fraction was detected, due to the high concentration of FS present in MC, representing 0.65% of the total solids in 30 mL of MC. The value ​​of 86.8% represented the non-biodegradable FS fraction (minerals), along with the FS fraction from the oat (Table 2). Antarctic soil has an extremely high fraction of fixed solids/minerals, as it contains low amounts of organic carbon (possibly including plant biomass, marine inputs, ornithogenic deposits, and endolithic communities), much of which is fixed or stabilized in mineral aggregates due to the extremely cold and dry conditions (da Silva et al. 2025).

In tests with the VS, the IO sample reached 96.58%, demonstrating high potential for biogas generation. A similar result was obtained for the same type of crushed sample, with 94.16% VS, while for uncrushed oats, the value was 92.66% VS (Dubrovskis and Plume 2017). These results show the importance of substrate pretreatment in improving biogas production. Using soil and sediment from different points on the Antarctic continent, including Elephant Bay, Livingston Island, Robert Island, and O’Higgins Station on the Antarctic Peninsula, Aguilar-Muñoz et al. (2022) observed a very low percentage of VS, reaching 1.2 to 5.5%. The present study showed a higher volume of VS in the MC and O + MC samples, reaching 13.2% and 86.11%, respectively, demonstrating that microbial enrichment from the collected soil and the addition of organic material (oats) provided a slight improvement in the biogas production potential.

### Biochemical Methane Potential (BMP) and methanogenic microbial diversity

For results to be reliable, cellulose must have a minimum production of 80% (VDI 4630 2016). Cellulose production reached 684 LN kg VS⁻^1^ of biogas, which represents 91,2% of the established value (data not shown).

The results of the assays with IO and O + MC were 595 LN kg VS⁻^1^ and 561 LN kg VS⁻^1^ of biogas, respectively. Higher values compared to work using only oat and cattle manure-based inoculum, reaching 400 LN kg VS⁻^1^ and 439 LN kg VS⁻^1^, respectively, without the addition of an enriched MC (Dubrovskis and Plume 2017). The methane produced reached 53% and 57% in the assays with IO and O + MC, respectively. A 4% increase in methane production when optimized microbial enrichment of Antarctic soil was used. A better methane production compared to the study performed by Dubrovskis and Plume (2017), where the authors observed 48% and 44% methane produced with crushed and uncrushed oats, respectively, without the use of microbial enrichment (Table 2).

Factors such as the composition of the inoculum and the substrate/inoculum ratio (S/I) can influence the biogas production. A ratio lower than 2:1 (S/I) allows for adequate performance in anaerobic digestion; on the other hand, higher values may destabilize the process due to acidification and the accumulation of VFAs (Alexis et al. 2015). At low temperatures, biogas production decreases significantly due to reduced viability of methanogenic communities, which are more active at mesophilic or thermophilic temperatures. Wang et al. (2019) observed a decrease in biogas production (between 10—70%) with a reduction in temperature range from 30–20 °C. In the study, methane represented 57% of the total biogas production at 35 °C; however, when the temperature dropped from 25 °C to 20 °C, the methane content decreased considerably, reaching only 25%. Thus, the use of microbial enrichment from a cold environment, even when cultivated at a temperature close to the mesophilic range (28 °C), can improve methane production in an AD.

**Fig. 2:**
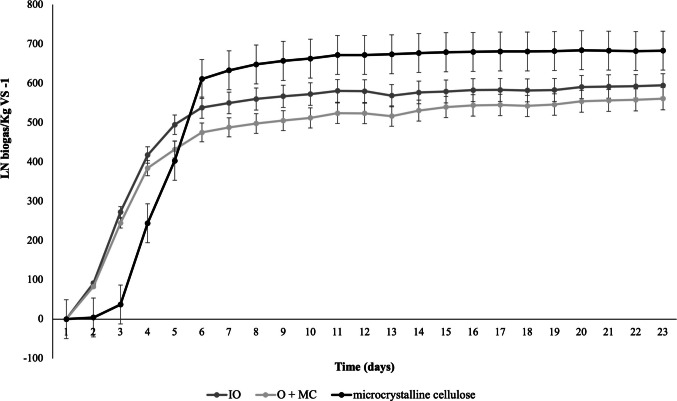
Average cumulative biogas production in the BMP test

The assay was performed until the thirty-first day of AD, since the measured volume became 0.5% lower than the total accumulated volume (VDI 4630 2016). It can be observed that biogas production in both samples increased exponentially until approximately the tenth day, which may be associated with the consumption of most of the available organic matter up to that point (Fig. 2). The maximum biogas production of the O + MC assay was lower than that obtained in the IO (Fig. 2). This difference may be associated with the increase of FS provided by the MC, which may be represented by minerals present in the Antarctic soil (da Silva et al. 2025), mainly on Deception Island, due to volcanic activity. According to Centurion et al. (2019), geothermal activity on Deception Island may be responsible for high levels of heavy metals. The presence of heavy metals can significantly affect microbial activity, thus reducing the microbial structure and composition of the soil (Campillo-Cora et al. 2025). Therefore, it can hinder microbial activity and decrease biogas production. The statistical comparison of TS, VS, FS, biogas, and methane production between treatments revealed no significant difference between the samples (Kruskal–Wallis: H = 1.692; ANOVA F = 1.14). All p-values ​​were > 0.05.

Most of the studies on bioprospecting and application of methanogenic microorganisms in cold environments used samples from lakes and permafrost in the Northern Hemisphere (Olivera 2022; Thalasso et al. 2022) and explored the diversity of communities and methanogenic activity, addressing molecular methods or anaerobic tests in the presence of anaerobic digestion substrates such as H_2_ and acetate (Rafael 2018). A study demonstrated methane production by microbial cells isolated from enrichment cultures sampled from an Antarctic Lake covered with perennial ice (Li et al. 2020). Another study assessed methane production directly in samples collected from two tundra marshes in East Antarctica. CH_4_ fluxes for the wet and mesic tundra sites averaged 163.4 μg m^−2^ h^−1^ and for the mesic sites 132.4 μg m^−2^ h^−1^. At dry sites, all CH_4_ fluxes were negative. Flux measurements in lakes and marshes were 170.4 μg m^−2^ h^−1^ and 134.7 μg m^−2^ h^−1^, respectively (Zhu et al. 2007). AD using microorganisms from Deception Island has not yet been reported. A study on microbial diversity samples from Deception Island, at Whalers Bay and Fumarole Bay, reported unique archaeal diversity, showing different survival strategies, especially those related to the Nanoarchaeota, and thermophilic archaea (Bendia et al. 2021).

**Fig. 3:**
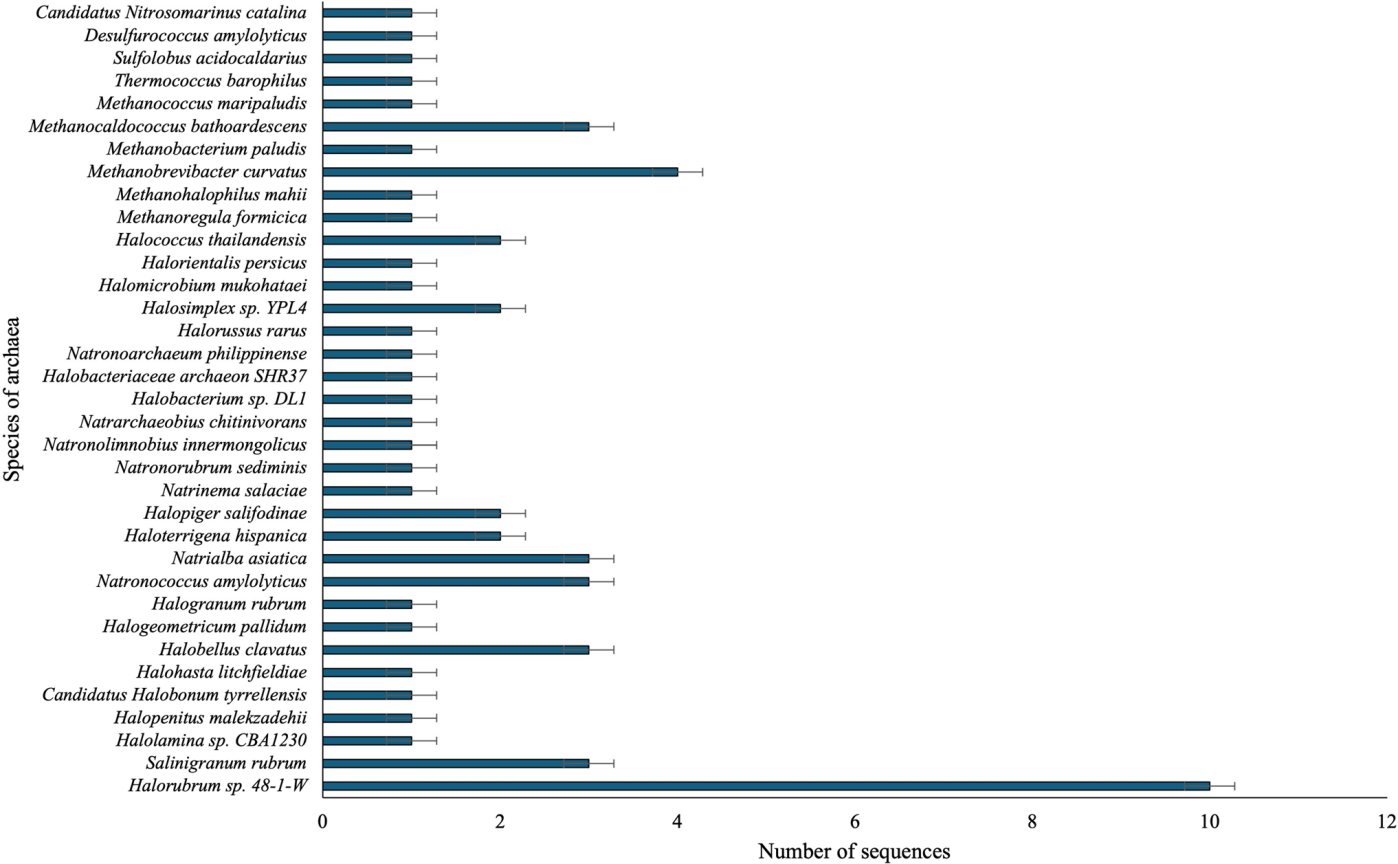
Taxonomic distribution of archaeal species in the thawed soil

According to Passarini et al. (2024), after processing the sequencing data, 94.648 thousand high-quality DNA sequences from 113.484 thousand raw sequences were recovered. This shallow sequencing depth may have affected the interpretation of the archaea diversity associated with the studied soil sample. In the present work, a total of 25.852 genera were annotated, 61 were associated with the Archaea domain, representing a relative abundance of approximately 0.23% (Fig. 3**)**. Metagenomic analyses showed a low archaeal diversity and abundance in an oligotrophic soil found on the Antarctic continent. Thirty-five species from the phyla Euryarchaeota (95.2%), Crenarchaeota (3.2%), and Thaumarchaeota (1.6%) were recovered. Regarding methanogenic groups, 7 genera, including *Methanoregula*, *Methanohalophilus*, *Methanobrevibacter*, *Methanobacterium*, *Methanocaldococcus*, *Methanococcus,* and *Thermococcus*, were observed (Fig. 3).

Methanogenic groups can be found in various substrates of the Antarctic continent. In subglacial lake sediments, the phylum Euryarchaeota was found, including the genera *Methanohalophilus* and *Methylobacter*. Archaea in deep-sea sediments are represented by Thaumarchaeota (Doytchinov and Dimov 2022; Stibal et al. 2012). In a study conducted with enriched permafrost in the Antarctic Dry Valley, the authors observed methane production associated with the genus *Methanosarcina* and cold-adapted enzymatic metabolic pathways (Vishnivetskaya et al. 2018).

Evaluating the temperature in anaerobic digestion processes, Aguilar-Muñoz et al. (2022) observed methane production between 5 and 37 °C. Higher production was observed at 25 °C, associated with the relative abundance of methanogenic archaea, including *Methanosaeta* (5 and 25 °C), and *Methanocorpusculum* (30 °C). In a study using permafrost deposits from King George Island (Antarctica), the authors observed the phylotypes *Methanobrevibacter* and *Methanosarcina*, which utilize the hydrogenotrophic, acetoclastic, and methylotrophic methanogenesis pathways (Karaevskaya et al. 2014).

Exploring the diversity of Archaea in lake sediments on James Ross Island, Antarctica, a low taxonomic diversity of methanogens was observed, not exceeding 1% of the total prokaryotic community, with the genera *Methanothrix* and *Methanosarcina* being the predominant methanogens in the lake samples and the first report of *Methanothermobacter* sp. in Antarctica (Buriánková et al. 2022). Some of these previous studies corroborate our findings, with the presence of representatives of the same methanogenic genera (*Methanohalophilus* and *Methanobrevibacter*) in the soil sample collected on Deception Island. The presence of hydrogenotrophic methanogens, even in low abundance, including the genera *Methanoregula*, *Methanobrevibacter*, *Methanobacterium*, *Methanocaldococcus*, and *Methanococcus* (Siegert et al. 2015), which can utilize H_2_ and CO_2_ to produce methane, may explain the slight methane production in the O + MC samples. The metabolic pathways of methane were based on taxonomic inferences and were not evidenced by the presence of functional genes.

Soil from the Antarctic continent, methanogenic communities can be active even at higher temperatures, including 28 °C (temperature used in the enrichments) and 37 °C (temperature used in the BMP assay). In this way, soil samples from the Antarctic continent can be used in future research innovations as bioaugmentation strategies, employing temperature optimization in anaerobic digestion processes under cold conditions.

## Conclusion

The BMP results demonstrated that oats showed good potential for biogas production. Methane production using microbial enrichment from Antarctic soil improved the yield by 4% compared to the oat assay. Therefore, sustainable alternatives, such as bioaugmentation with soil enrichment from Whalers Bay, may be promising in anaerobic digestion studies using lignocellulosic residues at lower temperatures. However, further studies are needed, including low-temperature BMP assays, controlled anaerobic bioaugmentation experiments, the use of different culture media to better reproduce the Antarctic environment, sequencing of other soil samples, and functional metagenomics to address the limitations of this study.

## Data Availability

The datasets used and/or analyzed during the current study are available from the corresponding author on reasonable request.
